# A 6-month randomized controlled trial to test the efficacy of a lifestyle intervention for weight gain management in schizophrenia

**DOI:** 10.1186/1471-244X-13-60

**Published:** 2013-02-18

**Authors:** Cecília Attux, Larissa C Martini, Hélio Elkis, Sérgio Tamai, Andréa Freirias, Maria das Graças Miquelutti Camargo, Mário Dinis Mateus, Jair de Jesus Mari, André F Reis, Rodrigo A Bressan

**Affiliations:** 1 Department of Psychiatry, Universidade Federal de São Paulo. Rua Machado Bitencourt, 222, Vila Clementino, CEP 04044-000, São Paulo, (SP), Brazil; 2 Department and Institute of Psychiatry, University of São Paulo Medical School. Rua Dr. Ovídio Pires de Campos, 785 – Cerqueira César, CEP 05403010, São Paulo, (SP), Brazil; 3 CAISM (Centro de Atenção Integrada à Saúde Mental) from Irmandade Santa Casa de Misericórdia de São Paulo, Rua Major Maragliano, 287, Vila Mariana, CEP 04017030, São Paulo, (SP), Brazil; 4 Division of Endocrinology, Department of Medicine, Universidade Federal de São Paulo (UNIFESP). Rua Pedro de Toledo, 910, Vila Clementino, CEP 04039-002, São Paulo, (SP), Brazil; 5 Rua Borges Lagoa, 564 cj, 23 04038-000, São Paulo, SP, Brazil

**Keywords:** Schizophrenia, Clinical trial, Weight gain, Physical activity

## Abstract

**Background:**

Patients with schizophrenia have lower longevity than the general population as a consequence of a combination of risk factors connected to the disease, lifestyle and the use of medications, which are related to weight gain.

**Methods:**

A multicentric, randomized, controlled-trial was conducted to test the efficacy of a 12-week group Lifestyle Wellness Program (LWP). The program consists of a one-hour weekly session to discuss topics like dietary choices, lifestyle, physical activity and self-esteem with patients and their relatives. Patients were randomized into two groups: standard care (SC) and standard care plus intervention (LWP). Primary outcome was defined as the weight and body mass index (BMI).

**Results:**

160 patients participated in the study (81 in the intervention group and 79 in the SC group). On an intent to treat analysis, after three months the patients in the intervention group presented a decrease of 0.48 kg (CI 95% -0.65 to 1.13) while the standard care group showed an increase of 0.48 kg (CI 95% 0.13 to 0.83; p=0.055). At six-month follow-up, there was a significant weight decrease of −1.15 kg, (CI 95% -2.11 to 0.19) in the intervention group compared to a weight increase in the standard care group (+0.5 kg, CI 95% -0.42–1.42, p=0.017).

**Conclusion:**

In conclusion, this was a multicentric randomized clinical trial with a lifestyle intervention for individuals with schizophrenia, where the intervention group maintained weight and presented a tendency to decrease weight after 6 months. It is reasonable to suppose that lifestyle interventions may be important long-term strategies to avoid the tendency of these individuals to increase weight.

**Clinicaltrials.gov identifier:**

NCT01368406

## Background

The use of antipsychotic medications is essential for controlling the acute psychotic episode and to prevent relapse of schizophrenia, but they are associated with long-term weight gain [1]. Moreover, people with schizophrenia tend to practice little physical activity, are more prone to tobacco addiction and show preference for a diet rich in calories [2,3]. Such lifestyle, together with the side effects induced by antipsychotics, predispose individuals with schizophrenia be overweight or obese [1], with a higher risk for diabetes mellitus (DM) and cardiovascular diseases (CVD) [4], leading to a significant reduction in life expectancy [5,6]. Weight gain is also associated with the perception of poor quality of life, reduced general health, and low vitality [3], thus representing an important factor for non-adherence of medications [7]. Moreover, obese patients are more likely to discontinue their medication [8].

The need to provide lifestyle intervention programs to improve management of patients with schizophrenia is now widely recognized [9]. For instance, Faulkner et al. [10] conducted a review of pharmacological and non-pharmacological strategies for reducing or preventing weight gain in individuals with schizophrenia. They concluded that no single pharmacological agent is consistently superior in terms of weight-loss efficacy, and that non-pharmacological interventions which include dietary and physical activity modifications were effective to prevent weight gain.

Several lifestyle interventions have been tested for reducing the negative consequences of weight gain, and to decrease incidence and prevalence of DM and metabolic syndrome (MS) [11-14]. These lifestyles strategies for weight-gain management have been proven to be effective in clinical trials and include regular check-ups, lifestyle and medication counseling, medication assessments, behavioral control programs, and pharmacological intervention [10,15]. Most interventions used pharmacological adjuncts or cognitive behavioral interventions for reducing weight gain in patients with schizophrenia [10] though very few studies used lifestyle modifications for patients with schizophrenia [16]. Thus, the main aim of the present study was to test the efficacy of a 12-week group Lifestyle Wellness Program (LWP), as a strategy for weight gain management for individuals with schizophrenia.

## Methods

Lifestyle Wellness Program (LWP) is a 12-week weight management intervention developed by Eli Lilly Laboratories for controlling weight gain for individuals with schizophrenia under antipsychotic use [17]. The program consists of a one-hour weekly session to discuss topics like dietary choices, lifestyle, physical activity and self-esteem with patients and their relatives [18]. The program combines behavioural techniques such as the use of diaries and role play to dealing with stress, and psychoeducation components including awareness of dietary habits. The intervention is comprised by 12 sessions as follows: a) one session for the introduction of the intervention; b) four sessions for discussing dietary choices using the concept of the food pyramid; c) three sessions for discussing the importance of physical activity; d) one session for self-esteem and motivation; e) one session for management of anxiety; and f) one session opened to relatives, and h) the wrap up of the program [18]. The inclusion of relatives may be a particular feature of the program for countries where most of the patients live with their families as is the case in Brazil. The groups are led by mental health professionals (nurses, occupational therapists, psychologists and dietitians), who are trained with a manual and a set of DVDs explaining the program.

A multicentric randomized clinical trial was conducted to compare the efficacy of this Lifestyle Wellness Program (LWP) with controls on a standard care (SC) group. Patients on the intervention group and on standard care group had regular visits to the psychiatrist and attended regular sessions of other psychosocial interventions offered by the program they were enrolled. Participants were drawn from the following outpatient programs: a) the Schizophrenia Program (Programa de Esquizofrenia – PROESQ, Universidade Federal de São Paulo); b) the Schizophrenia Program of Institute of Psychiatry- PROJESQ (Universidade de São Paulo); c) the CAISM (Centro de Atenção Integrada à Saúde Mental) from Irmandade Santa Casa de Misericórdia de São Paulo; and d) the Psychosocial Community Center Luiz da Rocha Cerqueira, which is directed by the Universidade Federal de São Paulo, all located in the city of São Paulo.

Participants using any antipsychotic in the past three months, presenting a diagnosis on the schizophrenia spectrum confirmed by the Structured Clinical Interview for DSM-IV Axis I Disorders (SCID I-P) [19], aged between 18 and 65 years old, and being clinical stable, i.e., reaching less than 60 in the *Positive and Negative Syndrome Scale* (PANSS) scale [20] were asked to participate. They also needed to be motivated to lose weight or have showed some concern about weight gain. Participants were already enrolled in the outpatient units included in the study and were referred by either the clinician or a mental health worker of the team.

Patients were excluded if they were not clinically stable, in the presence of DM, or had a previous history of an eating disorder (Anorexia and Bulimia), or drug and alcohol abuse. Patients were not allowed to take any medication with the intention of controlling or reducing weight. Participants who agreed to take part in the study signed written informed consent and were randomly assigned to the intervention group or a standard care group using a randomization table available on the web site http://www.randomization.com. The protocol was submitted and approved by the Ethical Committee of each center.

The primary outcome was defined as weight and body mass index (BMI) changes. BMI was calculated as weight in kilograms divided by the square of the height in meters. Data on social and demographic characteristics, clinical data and physical examination (weight, height, BMI, waist circumference and blood pressure) were routinely recorded. Weight was recorded every month, in the morning, on the same scale (Kratos-cas Linea model), without shoes, with the individuals wearing light clothes. Waist was considered at the level of the navel. Blood pressure was measured twice, and the mean of both measures was considered. Measures were collected by the same investigator in all assessments.

Fasting plasma glucose, insulin, total cholesterol, HDL-cholesterol, LDL- cholesterol and triglycerides levels were assessed at baseline, and at three- and six-month follow-up. A surrogate of insulin resistance, the Homeostatic Model Assessment (HOMA-IR) was calculated at baseline, at three and six months [21].

Blind investigators applied the following instruments to participants of the trial at baseline and three-month follow up: the *Positive and Negative Syndrome Scale* (PANSS) [20] to evaluate the severity of the disease, the Calgary Depression Scale [22] to assess depression, *Clinical Global Impression – Severity Scale (*CGI-S) [21] and *Clinical Global Impression- Improvement Scale (*CGI-I) [23] to assess clinical global impression. Global functioning was evaluated by *Global Assessment of Functioning* (GAF) [24], and independent living skills by *Independent Living Skills Survey*- patient version (ILSS-BR/P) [25]. Patients were asked to reply to the following self-rated scales: The World Health Organization's WHOQOL-BREF quality of life assessment- WHOQoL-BREF [26], Rosenberg self-esteem scale [27], Dietary Instrument for Nutrition Education (DINE) to classify dietary fat (satured and unsatured fat) and fiber intakes [28], Fagerström tolerance questionnaire to evaluate tobacco dependence [29], and International Physical Activity Questionnaire-short version (IPAQ) [30] to evaluate physical activity. IPAQ short form is an instrument designed primarily for population surveillance of physical activity among adults. IPAQ classifies physical activity into three categories: walking, moderate and vigorous activity.

Patients were evaluated at five moments during the study: baseline (physical examination, blood tests and scales), at one-month and two-month follow up (weight and BMI), at three-month follow up (physical examination, blood tests and scales), and at six-month follow up (physical examination and blood tests). No inputs about lifestyle were given after the 12-week program.

### Sample size

The sample size was estimated based in an open pilot study with 48 patients, where it was showed a weight difference of -1 kg (weight loss) and a standard deviation of 2 after three months. Taking into account a 35% of drop-outs (α=0.05, power 0.8), the expected number in each group was found to be 90 patients.

### Statistical analysis

All randomized subjects were included in the initial analysis. Weight and BMI changes were defined as main outcomes. Two-sided t-tests and chi-square tests were used to analyze the differences between the groups at baseline and during follow-up.

ANOVA with repeated measures was used to compare the intervention versus standard care groups over time. We describe two types of p value, one that represents difference over time and the other that represents interaction between groups. Analyses of the main outcomes were based on the intention-to-treat analysis, with the Last Observation Carried Forward (LOCF) using weight and BMI measures of the last assessment available. An alpha level of .05 was set for all statistical tests. Data was analyzed using Statistical Package for Social Sciences, version 15 (SPSS Inc, Chicago, Illinois).

## Results

### Randomization and participant characteristics

**Figure 1:**
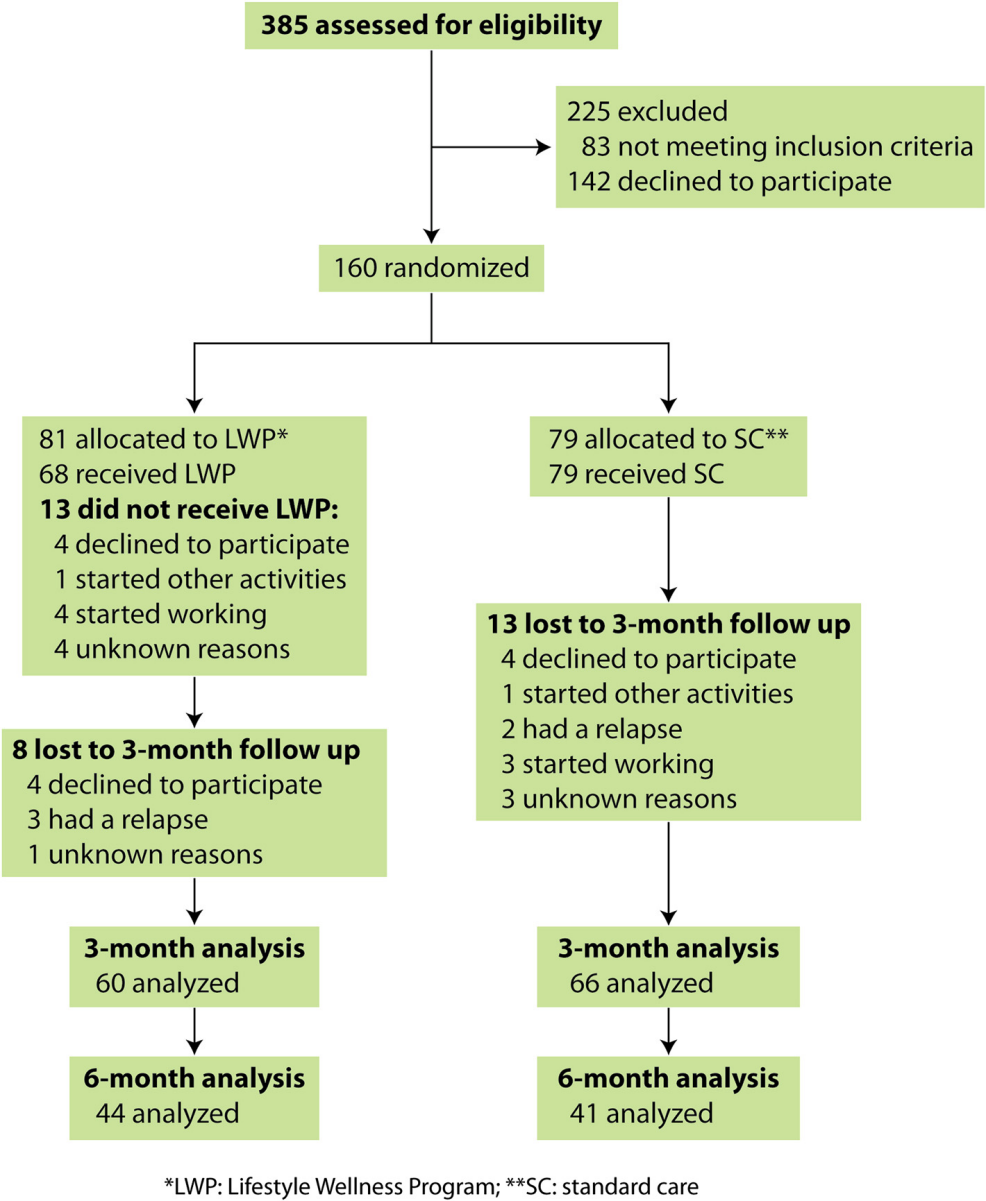
CONSORT diagram: participation in the study.

A total of 160 patients were included in the study, 81 were randomly allocated to the Lifestyle Wellness Program (LWP) and 79 patients to the standard care (SC) group. Figure 1 shows the CONSORT diagram of events among participants of the study. Overall there were 34 dropouts for the three-month follow-up. Thirteen patients assigned to the LWP group did not attend any of the sessions as follows: a) four patients declined to participate; b) one patient started new activities at the same time of the group; c) four patients started working in the beginning of the intervention; and d) four patients were missed for unknown reasons. During the three-month follow-up there were 8 dropouts in the LWP intervention group (4 declined to participate, 3 had a relapse, 1 missed for unknown reasons) and 13 in the standard care (4 declined to participate, one started other activities, 2 had a relapse, 3 started working, and 3 were missing for unknown reasons), leading to a response rate of 78.7%. At six-month follow up we had 44 patients in the intervention group and 41 patients in the standard care group, with a response rate of 53.1%.

As can be seen in Table 1, the social, demographic and clinical characteristics did not differ significantly between the two groups at baseline.

**Table 1:** The social, demographic and clinical characteristics of participants at baseline (n=160)

**Variable**	**Lifestyle wellness program (n= 81)**	**Standard care (n= 79)**	**p**
**Gender**			
**Female/Male**	31/50	33/46	0.747
**Age**	36.2 (9.9)	38.3 (10.7)	0.209
**Marital status**			
Single/Married/Other (divorced, widow)	64/8/9	62/10/7	0.787
**Ethnicity**			
Caucasian/Afro-American/other	60/11/10	58/15/6	0.444
**Education, years**			
1-8/9-11/>11 years	17/ 47/17	20/43/16	0.808
**Occupation**			
With/ without occupation	36/45	38/40	0.857
**Diagnosis (SCID)**			
Schizophrenia/ other psychosis	72/7	69/8	0.792
**Age of onset, years**	23.2 (9.0)	22.8 (8.7)	0.805
**Duration of illness, years**	13.1 (10.3)	15.5 (9.9)	0.142
**Antipsychotic drugs**			
First / Second generation/ Association /None	11/57/13/0	7/59/11/2	0.382
**Duration of current antipsychotic treatment, years**	5.1 (6.1)	4.0 (3.6)	0.209
**PANSS positive**	12.3 (5.1)	12.1 (4.5)	0.685
**PANSS negative**	16.7 (6.6)	18.7 (7.3)	0.133
**PANSS general**	27.4 (7.0)	30.7 (8.5	0.055
**PANSS total**	56.4 (15.1)	61.6 (17.5)	0.138
**Calgary**	2.6 (3.2)	2.8 (4.7)	0.233
**GAF**	60.4 (15.7)	58.9 (15.4)	0.596
**CGI**	3.2 (1.1)	3.4 (1.0)	0.571

### Intervention adherence

Patients participating in the program were followed up by the program coordinator who would call them in case of absence in the session. The mean of attending sessions was 9.1 (SD: 3.5), and 49 patients, i.e. 72.1% of participants attended eight or more meetings.

### Clinical and metabolic parameters at baseline

At baseline both samples were similar regarding clinical and metabolic parameters such as weight, BMI, waist measurement, blood pressure, blood glucose, total cholesterol, HDL-cholesterol, LDL-cholesterol, triglycerides, insulin, and HOMA-IR (p>0.05). The BMI (kg/m^2^) of the two groups showed presence of being overweight, and the SC group was slightly higher (29.9 kg/m^2^) than the intervention group (29.1 kg/m^2^). 117 patients (73.1%) were taking second generation antipsychotics (25 risperidone, 40 olanzapine, 34 clozapine, 10 quetiapine, 5 ziprasidone, 3 aripiprazole, 18 (11.4%) were taking first-generation antipsychotics (1 chlorpromazine, 3 thioridazine, 11 haloperidol, 4 other typical), and 24 patients (15.2%) were taking an association of antipsychotics. We found no difference between both groups regarding the type of antipsychotic (p=0.254).

### Changes over time

After three months, the intervention group (n= 60) presented a decrease of −0.47 kg (CI 95% -1.23 to 0.3) and the standard care group (n= 66) presented an increase of 0.46 kg (CI 95% -0.32 to 1.24), but this difference was not statistically significant (group interaction p=0.093).

The BMI of the intervention group showed a decrease of 0.14 kg/m^2^ (CI 95% -0.44 to 0.16) and the standard care group presented an increase of 0.16 kg/m^2^ (CI 95% -0.11 to 0.43; p=0.135).

After six months, the intervention group presented a decrease of 1.7 kg (CI 95% -3.17 to −0.23) and the standard care group showed an increase of 0.01 kg (CI 95% -0.51 to 1.45; p=0.099).

Blood glucose decreased in both groups over time after three months (p=0.029), however the decrease was not statistically different between the two groups.

Both groups presented an increase in walking as measured by IPAQ walking (p=0.002), as well as an increase in psychological domain of WHO-QoL quality of life scale (p=0.014). However the increase was not statistically different between the two groups on both scales.

### Changes between groups

After three months there were no differences between groups in waist, blood pressure, total cholesterol, HDL-cholesterol, LDL-cholesterol, triglycerides, insulin and HOMA-IR (Table 2).

**Table 2:** Analysis of the clinical characteristics at baseline and after three month follow-up

**Variables**	**Lifestyle wellness program (n=60)**		**Standard care (n=66)**		**p**
	**Baseline**	**3-month**	**Baseline**	**3-month**	
Weight (kg)	81.1 (14.3)	80.7 (14.1)	84.3 (17.8)	84.7 (17.9)	0.093
BMI (kg/m^2^)	29.1 (4.7)	28.9 (4.7)	29.9 (5.2)	30.0 (5.2)	0.135
Waist (cm)	101.1 (11.2)	100.5 (11.0)	104.1 (13.6)	104.4 (14.1)	0.149
Systolic BP (mmHg)	115.7 (12.7)	114.1 (12.2)	118.3 (16.1)	115.6 (12.4)	0.655
Diastolic BP (mmHg)	78.1 (9.1)	75.9 (10.3)	78.6 (12.0)	78.8 (10.9)	0.304
Glucose (mg/dl)	96.6 (20.5)	94.3 (13.7)	101.2 (35.6)	96.9 (30.1)	0.497
Total Cholesterol (mg/dl)	197.7 (45.7)	191.7 (41.9)	197.1 (40.2)	194.2 (38.8)	0.513
HDL Cholesterol (mg/dl)	44.2 (13.0)	44.3 (12.6)	47.0 (13.7)	46.4 (11.9)	0.585
LDL Cholesterol (mg/dl)	120.2 (37.7)	115.4 (36.2)	115.7 (33.0)	113.3 (34.4)	0.562
Triglycerides mg/dl)	166.7 (95.6)	164.8 (85.1)	165.6 (102.7)	175.5 (112.9)	0.393
Insulin (μU/ml)	11.3 (10.5)	12.8 (12.5)	13.9 (12.1)	14.5 (10.3)	0.616
HOMA-IR	2.8 (3.1)	3.3 (5.1)	3.5 (3.3)	3.6 (3.2)	0.507

BMI: body mass index; BP: blood pressure; HOMA-IR: Homeostatic Model Assessment.

In addition there were no differences between groups in ILSS, WHOQoL, Fagerström, DINE and IPAQ scores (Table 3).

**Table 3:** Analysis of the secondary outcomes at baseline and after three month follow-up

**Variable**	**Lifestyle wellness program (n=60)**		**Standard care (n=66)**		**p**
	**Baseline**	**3-month**	**Baseline**	**3-month**	
**ILSS**	0.76 (0.1)	0.76 (0.1)	0.76 (0.1)	0.76 (0.09)	0.595
**WHOQoL**					
Physical	60.2 (15.9)	58.7 (15.4)	59.0 (16.6)	60.5 (17.4)	0.270
Psychological	54.9 (18.0)	58.4 (18.9)	58.4 (16.8)	61.1 (19.3)	0.736
Social relations	53.9 (23.4)	56.9 (22.2)	53.0 (25.5)	56.9 (24.5)	0.803
Environmental	54.8 (15.6)	57.1 (14.4)	55.8 (14.2)	56.5 (16.2)	0.489
**Rosenberg self esteem**	12.1 (4.3)	11.8 (4.8)	12.3 (5.0)	12.2 (5.0)	0.811
**Fagerström tolerance questionnaire**	6.3 (2.1)	6.4 (2.7)	6.3 (2.7)	6.3 (2.7)	0.331
**DINE**					
Fibers	40.1 (20.8)	44.4 (17.9)	42.6 (18.2)	42.4 (17.1)	0.211
Fat	32.0 (13.9)	30.2 (12.3)	34.7 (14.9)	37.2 (14.7)	0.126
Unsatured fat	9.8 (1.9)	10.1 (1.6)	10.0 (1.7)	10.3 (1.2)	0.944
**IPAQ**					
Walking	843.4 (1113.5)	1390.8 (2078.7)	670.6 (903.5)	1049.2 (1191.4)	0.410
Moderate activity	1006.7 (2021.8)	910.2 (1618.4)	1007.8 (2002.2)	1446.7 (3701.7)	0.503
Vigorous activity	750.6 (1677.8)	778.2 (1857.1)	559.5 (1626.2)	530.9 (1202.4)	0.873
Total	2591.7 (3258.4)	3116.6 (4234.1)	2314.4 (3167.9)	3088.5 (4787.2)	0.294

### Intent-to treat analysis

The intent-to treat analysis was conducted only to weight differences since it was our primary outcome. The intent-to treat analysis included 146 patients, with a dropout rate of 8.75%.

After three months patients in the intervention group presented a decrease of 0.48 kg (CI 95% -0.65 to 1.13) while the standard care group showed an increase of 0.48 kg (CI 95% 0.13 to 0.83; p=0.055).

After six months the intervention group presented a decrease of 1.15 kg (CI 95% -2.11 to 0.19) and the standard care group presented an increase of 0.5kg (CI 95% -0.42 to 1.42), and this difference was statistically significant (p=0.017).

## Discussion

To our knowledge the present study is the largest randomized clinical trial designed to evaluate the efficacy of a lifestyle intervention (LWP) for weight gain management in patients with schizophrenia and schizoaffective disorders. At the end of the intervention (three months) there was no significant difference between groups on weight and BMI, or other metabolic parameters. However, after six months, patients who received LWP had lost 1.15kg and the patients under SC had gained 0.5 kg, and this difference was statistically significant, although the magnitude of the difference was small and not clinically significant. Therefore, the intervention group maintained weight and presented a tendency to decrease weight after 6 months.

On a recent systematic review, Álvarez-Jiménez et al. [16] reported ten different types of studies with non-pharmacological interventions lasting from eight weeks to six months to reduce weight gain in patients with schizophrenia. Six of these studies included trials with cognitive behavioral therapy, 3 nutritional counseling and only one a combination of nutritional and exercise interventions. Overall they found a significant weight reduction when intervention groups were compared to treatment as usual and the magnitude of this reduction was 2.56 kg (CI −3.2 to −1.92 kg, p< 0.001). There were no differences between the types of intervention.

Some studies were designed to target patients who had already gained weight. For instance, Kwon et al. [31], evaluated 48 patients comparing a 12-week individual lifestyle intervention with standard care, and found weight loss of 3.9 kg in the intervention group compared with 1.48 kg in the control group after three months (p<0.05). Wu et al. [32] in a randomized controlled trial with 128 patients in first episode schizophrenia comparing placebo, metformin, lifestyle intervention and metformin with lifestyle for weight gain management, found that lifestyle intervention and metformin alone and in combination demonstrated efficacy for antipsychotic-induced weight gain. Lifestyle associated with metformin showed the best effect on weight loss.

Another observational study of a lifestyle intervention conducted with 373 patients from 49 Scandinavian cities (314 on intervention and 59 controls) [33] found a mean change of −0.5 kg (95% CI: -0.9;-0.2) in weight lost for the intervention group and 0.9 kg (95% CI: 0; 1.8) increase for the control group, after three months follow-up, very similar findings with this clinical trial.

In this study there were no statistically significant differences between the groups for clinical and laboratory parameters associated with obesity. Blood glucose decreased in both groups over time differently from other studies that found an impact of intervention on glucose and lipid profile [34,35].

Pharmacological interventions for weight loss in schizophrenia have to take into account the risk of these medicines exacerbating psychotic symptoms [10]. Lifestyle interventions are safer and effective for promoting decrease or maintenance of weight. In a recent systematic review of effectiveness of treatments for obesity in adults Le Blanc et al. [36] found that behaviorally based treatment resulted in 3-kg greater weight loss in intervention than control participants after 12 to 18 months, with more treatment sessions associated with greater loss [36]. Controls generally lost little or did not gain weight, whereas intervention groups lost 1.5 to 5 kg, an average of 4% of the baseline weight [36]. It is noteworthy that weight losses of as little as 5% in individuals at risk of metabolic syndrome may result in clinically meaningful reductions in morbidity and risk of early mortality [37]. In addition, interventional studies that have achieved approximately 5% reduction in body weight together with increasing physical activity to at least 150 min/week of moderate activity such as walking, have resulted in a marked decreased in insulin resistance and a major reduction (50-80%) in the risk for future DM type 2 [11,13].

Limitations of this study include the short duration of the interventions and follow up. Although longer interventions are more appropriate for weight loss programs, most of the studies reported so far were conducted with a 12-week follow-up, similar to the current study [31,38]. The number of participants in the trial was slightly lower (160) than the estimated sample size (180). As the SC showed a significant increase in physical activity (walking), it is not possible to disregard some contamination. This lifestyle intervention had previously been conducted at these sites selected for the study, and this fact may have influenced the staff’s attitudes owed to increased awareness of physical health monitoring. This problem does not affect the main findings of the study, since it would contribute to increasing the efficacy in the control group, decreasing the odds of finding a significant difference with the intervention group. It is noteworthy that motivation for losing weight was part of the inclusion criteria for the study added to the fact that these patients were under care of programs directed by preeminent academic departments in the country, where it is supposed to expect some sort of intervention for losing weight in the control group. As health behaviors assessed in the study were not different between the two groups it is unclear the trajectory for weight change improvement in the experimental group.

As for the motivation factor it is worth noting that the intervention has a minor impact on weight change in the experimental group. Most of the studies did select motivated individuals to lose weight because this is an important factor of compliance. This intervention may not work for those who have no intention to change their lifestyle.

Weight management interventions for individuals with severe mental disorders should be incorporated to clinical practice since it is known the impact of obesity in general health and its consequences as diabetes and cardiovascular disorders. Lifestyle Wellness Program (LWP) can be an interesting option due to the fact that it is an easy and accessible intervention, which can be incorporated on routine of community services and outpatient facilities. Moreover, the cost of implementing such intervention can be very low once it can be delivered by non-specialist health workers of the existent health team and in the same setting of the community center.

## Conclusion

This was a multicentric randomized clinical trial with a lifestyle intervention for individuals with schizophrenia, where the intervention group maintained weight. It is reasonable to suppose that Lifestyle Interventions may be important long-term strategies to avoid the tendency of these individuals to increase weight. These group interventions can be delivered at a low cost, are safer than employing weight loss medicines, and may have long-term impact on quality of life and increased longevity.

## Competing interests

Cecília Attux: Fundação de Amparo a Pesquisa do Estado de São Paulo (FAPESP), Eli Lilly do Brasil: research funding; CAPES (Coordenação de Aperfeiçoamento de Pessoal de Nível Superior), the Ministry of Education: scholarship; Janssen-Cilag, Novartis, Roche, Eli Lilly do Brasil honoraria/ travel support. Larissa C. Martini: Eli Lilly do Brasil: honoraria/travel support; Astra Zeneca and Janssen-Cilag honorária; CAPES (Coordenação de Aperfeiçoamento de Pessoal de Nível Superior), the Ministry of Education: scholarship. Helio Elkis: Fundação de Amparo a Pesquisa do Estado de São Paulo (FAPESP), Janssen-Cilag, Novartis, Roche: research funding; Astra Zeneca, Janssen-Cilag, Eli Lilly do Brasil, Novartis: honoraria/ travel support; Astra Zeneca, Janssen, Pfizer: advisory board; Astra Zeneca, Janssen-Cilag, Merck-Sharp Dome, Novartis: speaker’s bureau.Sérgio Tamai: Pfizer: honoraria/ travel support. Jair de Jesus Mari: Fundação de Amparo a Pesquisa do Estado de São Paulo (FAPESP), CAPES, and National Research Council (CNPq): research funding; Janssen-Cilag, Astra Zeneca, and Eli Lilly do Brasil: speaker’s bureau. André F. Reis, Andrea Freirias, Maria das Graças M. Camargo, Mário Dinis Mateus: none. Rodrigo A. Bressan: Fundação de Amparo a Pesquisa do Estado de São Paulo (FAPESP), National Research Council (CNPq), CAPES, Fundação Safra, Fundação ABRADS, Janssen-Cilag, Novartis, Eli Lilly do Brasil, Lundbeck, Roche: research funding; Astra- Zeneca, Bristol, Lundbeck, Revista Brasileira de Psiquiatria: speaker/board; Radiopharmacus Ltda., Biomolecular Technology Ltda: shareholder.

## Authors' contributions

CA had full access to all of the data in the study and takes responsibility for the integrity of the data and the accuracy of the data analysis. It also participated in the study design, collection, analysis and interpretation of data, drafted the manuscript, and performed the statistical analysis, approval and drafting of final report. LCM participated in the administrative, technical and material support, collection, analysis and interpretation of data, drafted the manuscript, participated in the statistical analysis, approval and drafting of final report. HE participated in the analysis and interpretation of data, critical revision of the manuscript for important intellectual content and final approval and drafting of final report. ST participated in the analysis and interpretation of data, critical revision of the manuscript for important intellectual content and final approval of final report. AF participated in the acquisition of data, administrative and technical support and final approval of final report. MGMC participated in the acquisition of data, administrative, technical, or material support and approval of final report. MDM participated in the analysis and interpretation of data, critical revision of the manuscript for important intellectual content and approval of final report. JJM participated in the study concept and design, analysis and interpretation of data, drafting of the manuscript, critical revision of the manuscript for important intellectual content, approval and drafting of final report and study supervision. AFR participated in the study concept and design, analysis and interpretation of data, critical revision of the manuscript for important intellectual content, approval and drafting of final report and study supervision. RAB participated in the study concept and design, obtained funding, analysis and interpretation of data, statistical analysis, drafting the manuscript, critical revision of the manuscript for important intellectual content, approval and drafting of final report study supervision. All authors read and approved the final manuscript.

## Pre-publication history

The pre-publication history for this paper can be accessed here:

http://www.biomedcentral.com/1471-244X/13/60/prepub
